# Liver biopsy in the modern era: from traditional techniques to artificial intelligence and multi-omics integration

**DOI:** 10.3389/fmed.2025.1678753

**Published:** 2025-11-24

**Authors:** Nasar Alwahaibi, Maryam Alwahaibi

**Affiliations:** Department of Biomedical Science, College of Medicine and Health Sciences, Sultan Qaboos University, Muscat, Oman

**Keywords:** liver biopsy, artificial intelligence, multi-omics, hepatology, digital pathology, histological quantification

## Abstract

Liver biopsy remains a cornerstone in the diagnosis and management of various hepatic disorders. This mini-review provides a concise overview of traditional liver biopsy techniques, percutaneous, plugged, transjugular, and laparoscopic, highlighting their clinical indications, histopathological evaluation, and limitations. The review also explores recent advancements, including the integration of artificial intelligence in imaging modalities such as ultrasound, MRI, and CT, as well as its emerging role in histopathological analysis, particularly for assessing fibrosis, steatosis, inflammation, and cancer. In parallel, the application of multi-omics technologies is discussed as a promising complement to histology, offering molecular-level insights into disease progression and therapeutic response. Despite these technological steps, there remains a gap in the literature regarding how traditional biopsy methods are being effectively integrated with these emerging tools, and how liver biopsy continues to retain its clinical relevance in the era of artificial intelligence and multi-omics approaches. This review underscores the evolving landscape of liver biopsy and calls for harmonized frameworks that combine conventional techniques with digital innovations to enhance diagnostic accuracy, standardization, and patient care.

## Introduction

Liver biopsy remains a cornerstone in the diagnosis and management of various hepatic disorders, offering direct histological assessment that guides prognosis and therapeutic decisions (1). Despite advances in non-invasive modalities such as transient elastography and serum biomarkers, biopsy continues to provide unmatched specificity in evaluating disease activity, staging fibrosis, and identifying comorbid conditions (2). Traditionally performed through percutaneous access, liver biopsy has evolved to include transjugular, laparoscopic, and image-guided approaches, each with unique indications, benefits, and limitations (3).

Recent years have witnessed the development of innovative technologies aimed at improving diagnostic yield, minimizing complications, and expanding the applicability of liver biopsy in challenging clinical scenarios (4). These include real-time ultrasound-guidance, contrast-enhanced imaging, robotic assistance, and refinements in needle design (5). At the same time, growing awareness of procedural risks such as bleeding and pain, has led to stricter procedural criteria and standardization efforts to ensure safety and sample adequacy (6, 7).

This mini-review summarizes both traditional and modern liver biopsy techniques, highlighting current practices, evolving innovations, and emerging trends. It also discusses the role of liver biopsy within the broader context of hepatology, particularly as non-invasive alternatives continue to gain prominence. By examining these developments, we aim to provide a concise overview for clinicians, pathologists, and researchers engaged in liver disease diagnostics. However, there remains a gap in how traditional methods integrate with emerging technologies and how liver biopsy continues to retain its relevance in the era of artificial intelligence and multi-omics approaches. This revised mini-review emphasizes the transformative role of AI and multi-omics in redefining the clinical and research relevance of liver biopsy. Rather than listing individual algorithmic approaches, the focus is on conceptual integration, how digital pathology and molecular analytics can together enhance the interpretive and prognostic power of histological assessment.

## Traditional liver biopsy techniques

The conventional methods for liver biopsy have been well-established for decades, with the percutaneous approach remaining the most commonly employed technique (8). This method involves inserting a core needle, usually under local anesthesia, through the right lower intercostal space to access the liver parenchyma (9). Historically performed blindly, the percutaneous biopsy is now often image-guided using real-time ultrasound to enhance safety and accuracy (10). Percutaneous liver biopsy is indicated in patients with diffuse liver disease where coagulopathy is absent and the liver is easily accessible. It provides sufficient tissue for histological evaluation in most cases but carries risks such as pain, bleeding, bile leak, and, rarely, hemothorax or pneumothorax (11). Sample adequacy depends on the length and number of complete portal tracts, with at least 11 portal tracts being considered optimal and measured 20–25 mm long for diagnostic accuracy (12).

A plugged liver biopsy is a variation of the percutaneous technique designed for patients at high risk of bleeding, such as those with coagulopathy or thrombocytopenia. While a transvenous biopsy is also an option for these patients, the plugged approach is preferred when a larger tissue sample is needed. The procedure follows the standard percutaneous method, but differs in that the biopsy tract is sealed with materials like gel foam, collagen, or thrombin as the sheath is withdrawn to minimize bleeding (13).

**FIGURE 1:**
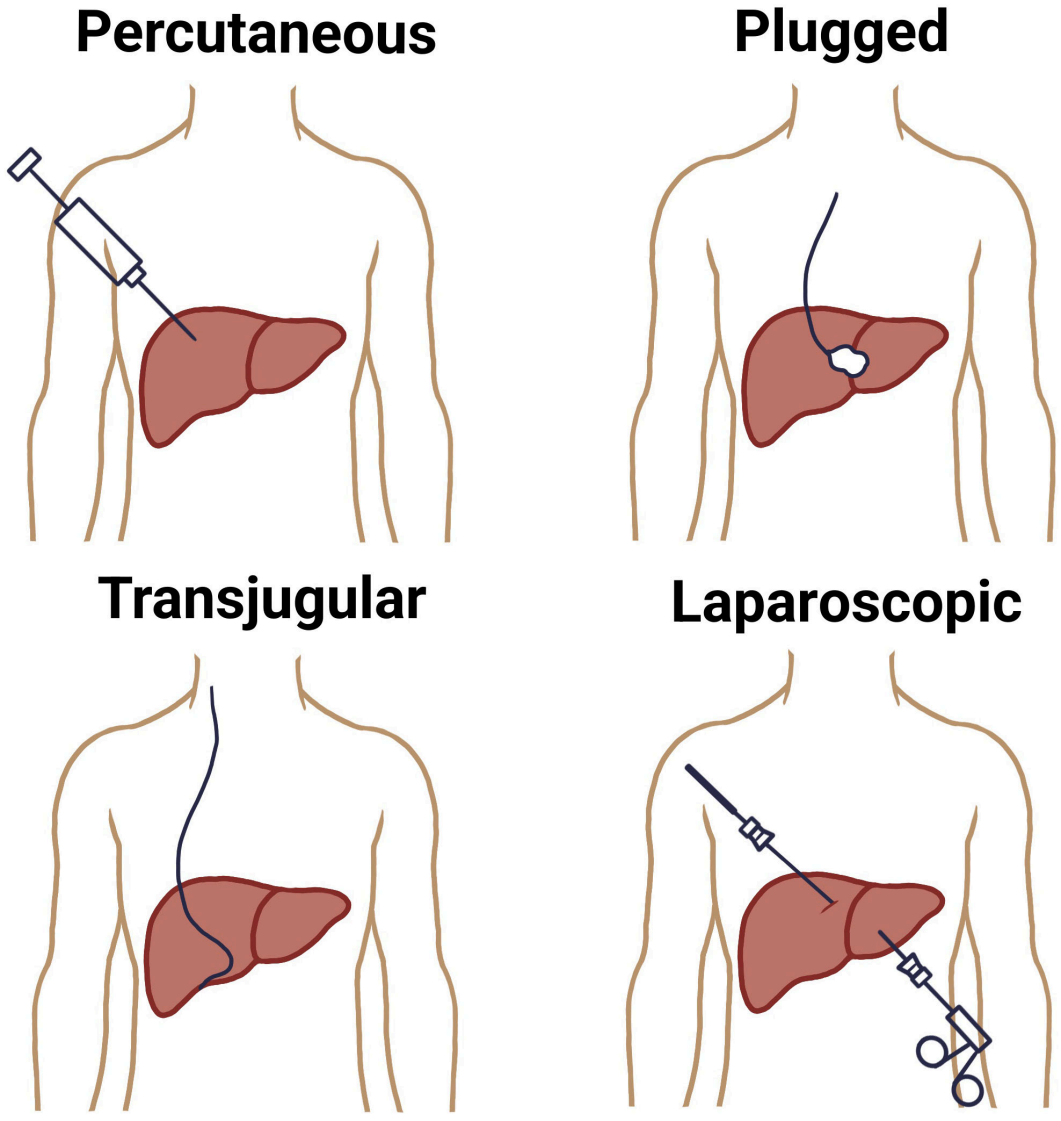
Different liver biopsy techniques.

When percutaneous biopsy is contraindicated, especially in patients with coagulopathy, thrombocytopenia, or massive ascites, the transjugular liver biopsy (TJLB) offers a safer alternative (14, 15). This technique involves catheter-based access to the hepatic vein via the internal jugular vein, allowing for intravascular sampling without traversing the liver capsule. Though technically more complex and associated with a smaller sample size, TJLB enables pressure gradient measurements and is preferred in patients with suspected vascular liver diseases or advanced portal hypertension (16). A total of 3–4 core samples are recommended to ensure an adequate number of complete portal tracts (CPTs) for reliable pathological evaluation (17). Another traditional modality, the laparoscopic liver biopsy, is performed under general anesthesia and provides direct visual access to the liver surface, permitting targeted sampling of focal lesions or suspicious nodules (18). It is frequently combined with other diagnostic or therapeutic procedures and is particularly useful in cases of focal liver lesions or when prior percutaneous biopsies have yielded non-diagnostic samples (19, 20) (Figure 1).

## Indications for liver biopsy

A liver biopsy is a valuable diagnostic and management tool used in various clinical scenarios to provide essential information about liver health and disease (21). There are many indications for performing a liver biopsy. When non-invasive tests (such as blood tests, imaging studies, or clinical evaluations) are inconclusive, a liver biopsy can provide definitive histological evidence to confirm or clarify the diagnosis of liver disease. This is particularly useful in conditions such as autoimmune hepatitis, metabolic liver diseases (e.g., Wilson’s disease, hemochromatosis), cryptogenic liver disease, and drug-induced liver injury (22). A liver biopsy offers a direct assessment of the extent and severity of liver damage. It helps to stage fibrosis, assess the level of inflammation, or identify cirrhosis in patients with chronic liver diseases like viral hepatitis (e.g., hepatitis B or C), metabolic dysfunction-associated steatotic liver disease (MASLD) (23, 24).

For patients with liver masses or tumors [e.g., hepatocellular carcinoma (HCC), metastatic cancers, or rare liver tumors], a biopsy allows for histopathological analysis to determine the type, grade, and nature of the tumor, which is critical for establishing an accurate diagnosis and planning appropriate treatment (25, 26). In individuals with an established liver condition, a biopsy provides detailed information about the progression or current state of the disease. This can help predict the likelihood of complications, such as liver failure or cancer, and inform discussions about prognosis with patients (27).

The findings from a liver biopsy can guide therapeutic interventions by identifying disease severity, type, or specific characteristics. For example, in autoimmune hepatitis, a biopsy may confirm the need for immunosuppressive therapy, while in metabolic conditions, it may highlight the urgency of specific treatments (e.g., chelation therapy in Wilson’s disease) (28). For chronic liver diseases, a biopsy may be performed to evaluate how the condition is progressing or to assess the effectiveness of treatments. For instance, in hepatitis C patients undergoing antiviral therapy, a biopsy can reveal whether liver inflammation and fibrosis are improving (29). Beyond histological examination, liver tissue obtained through biopsy can be used for microbiological, biochemical, or other specialized analyses. This is particularly important in cases where infections (e.g., fungal, bacterial, or parasitic) or metabolic abnormalities are suspected and require detailed investigation (30). By providing critical diagnostic and prognostic information, liver biopsy remains an indispensable tool in modern hepatology, even as non-invasive diagnostic tools continue to evolve. However, the decision to perform a liver biopsy should always be carefully weighed against the potential risks and benefits for each individual patient.

## Histopathological evaluation

Histopathological diagnosis of liver biopsy relies on microscopic examination of tissue sections stained with routine and special histological stains to evaluate liver architecture and cellular changes. Hematoxylin and eosin (H&E) method is the standard stain used for assessing general tissue morphology, including hepatocellular architecture, inflammation, necrosis, and fibrosis (31). Periodic acid–Schiff (PAS) stain highlights glycogen, basement membranes, and certain storage diseases, and when combined with diastase digestion (PAS-D), it helps identify α1-antitrypsin globules. Masson’s trichrome stain is employed to detect and grade fibrosis by highlighting collagen deposition in blue. Reticulin stain outlines the hepatic lobular framework, aiding in the evaluation of architectural distortion and regenerative nodules. Iron stains, such as Prussian blue, are used to detect hemosiderin deposition in conditions like hemochromatosis, while orcein can be used to identify copper-associated protein deposits in chronic cholestasis (32). Together, these stains provide a comprehensive assessment that supports the diagnosis and grading of various liver pathologies, including hepatitis, cirrhosis, metabolic diseases, and neoplasms.

## Liver biopsy scoring systems

To enhance consistency and reproducibility in histological interpretation, several standardized scoring systems are routinely applied in liver biopsy evaluation. The Kleiner scoring system is widely used for grading and staging non-alcoholic fatty liver disease (NAFLD), assessing steatosis, lobular inflammation, hepatocellular ballooning, and fibrosis (33). Similarly, the Ishak and METAVIR systems are commonly utilized for chronic viral hepatitis to quantify necroinflammatory activity and fibrosis stage [(34) 2010]. These structured frameworks enable uniform reporting, facilitate clinical trial comparisons, and support objective disease monitoring across different liver disorders.

## Limitations of traditional techniques

Despite their longstanding role in hepatology, traditional liver biopsy techniques, particularly percutaneous, transjugular, and laparoscopic, are associated with several limitations that can affect diagnostic accuracy, patient safety, and broader applicability. One of the most significant limitations is sampling error, due to the small size of tissue cores relative to the entire liver (35). This is particularly problematic in diseases with patchy or heterogeneous involvement, such as non-alcoholic steatohepatitis (NASH), autoimmune hepatitis, or focal nodular hyperplasia (36). A standard biopsy specimen represents less than 1/50,000 of the liver mass, raising concerns about understaging or misclassification of disease severity, especially fibrosis (37).

Histological interpretation relies on subjective assessment by pathologists, and significant interobserver variability exists, especially in grading inflammation or staging fibrosis in chronic hepatitis and NASH (38, 39). The lack of universally applied, objective scoring systems across centers contributes to inconsistent diagnosis and staging (40, 41). Traditional liver biopsy is an invasive procedure, associated with potential complications such as pain, bleeding, bile leak, injury to adjacent organs, and, rarely, death. Although the overall complication rate is low (∼0.5%), the perceived risks may discourage both clinicians and patients from proceeding, particularly when the diagnostic yield is uncertain. Technological advancements have considerably mitigated the procedural risks of liver biopsy. The introduction of real-time ultrasound and CT guidance has enhanced targeting accuracy, while the use of automated spring-loaded core biopsy devices and smaller gauge needles has significantly reduced bleeding and post-procedural pain. Together, these innovations have improved the safety profile and diagnostic yield of liver biopsy, transforming it into a more precise and patient-friendly procedure (42).

Certain clinical scenarios present technical limitations to biopsy. For example, coagulopathy, thrombocytopenia, or ascites may preclude percutaneous biopsy (43). While transjugular biopsy can be used in such settings, it requires fluoroscopy and specialized expertise, and often yields smaller, fragmented samples.

Repeated biopsies are often necessary to assess disease progression or treatment response, but their invasiveness and associated risks make longitudinal monitoring impractical. This restricts their utility in dynamic disease states or in the evaluation of new therapeutic interventions (44). While traditional histology provides valuable structural information, it offers limited insight into molecular pathways, immune responses, or gene expression profiles. These aspects are increasingly relevant in the era of personalized medicine and targeted therapies, where tissue-based molecular diagnostics are required to guide treatment (45).

## Clinical applications

Liver biopsy remains a critical tool in hepatology, offering unparalleled insights into liver architecture, pathology, and disease stage. Despite advancements in non-invasive diagnostics, tissue sampling is still required in many clinical contexts to confirm diagnosis, determine disease severity, and guide therapeutic decisions.

Liver biopsy remains a vital tool for both diagnosis and prognosis in liver disease, particularly when clinical or serologic tests are inconclusive. While infections like HBV and HCV can often be diagnosed through blood tests, many other liver conditions require histological evaluation alongside clinical and laboratory data. Biopsy is especially valuable in complex or overlapping presentations, such as differentiating autoimmune hepatitis (AIH) from MASLD, or evaluating coexisting disorders like steatosis with HCV or hemochromatosis (46–48). It is also critical for diagnosing hereditary and metabolic disorders (e.g., Wilson disease, alpha-1-antitrypsin deficiency, amyloidosis) (49–51), systemic diseases with hepatic involvement (52), and acute liver failure, where biopsy may reveal treatable conditions such as herpes infection, AIH, or malignancy (53). Prognostically, liver biopsy provides essential information through fibrosis assessment, which predicts progression to cirrhosis, portal hypertension, and liver-related mortality. This is particularly relevant in chronic HCV, where alcohol, hepatic iron, and steatosis accelerate fibrosis (54). Advanced fibrosis is linked to increased mortality in post-transplant HCV (37), and with worse outcomes in MASLD (55), and hemochromatosis, where cirrhotic patients face elevated risks of hepatocellular carcinoma (56). In AIH, cirrhosis at diagnosis indicates poorer prognosis (57). Furthermore, biopsy remains useful even in HIV/HCV patients with normal ALT, where it can reveal significant fibrosis with prognostic implications (58). Fibrosis regression is associated with fewer complications (59).

In the workup of indeterminate liver lesions, particularly when imaging is inconclusive, biopsy is invaluable. It enables histopathological classification of HCC, cholangiocarcinoma, metastatic tumors, or benign masses, guiding oncological management or surgical planning. In transplant candidates, liver biopsy can help detect incidental malignancy or underlying disease recurrence.

Liver biopsy is frequently performed in both pre- and post-transplantation settings to guide clinical decision-making (60). One of its key roles is in assessing transplant suitability, particularly during the evaluation of living donors and recipients with chronic liver disease of unclear etiology. In such cases, histological assessment provides critical information about the degree of fibrosis, steatosis, or other underlying pathology that may influence transplant eligibility and timing (61–66). In addition, liver biopsy is indispensable for monitoring graft function and detecting post-transplant complications. It plays a crucial role in differentiating among acute cellular rejection, recurrent or *de novo* liver disease, and drug-induced hepatotoxicity, all of which may present with overlapping clinical signs and biochemical abnormalities (67–70). Histological evaluation remains the most reliable method for distinguishing these conditions, enabling timely and appropriate therapeutic interventions that can significantly affect graft survival and patient outcomes.

Liver biopsy plays a pivotal role in clinical and translational research, offering access to liver tissue for detailed histological, molecular, and cellular analyses. It provides a unique opportunity to investigate disease mechanisms, validate emerging biomarkers, and assess therapeutic efficacy. In the study of pathogenesis, biopsy specimens are essential for exploring the underlying mechanisms of liver diseases such as MASLD, AIH, and viral hepatitis, allowing researchers to evaluate inflammation, fibrosis, steatosis, and cellular injury through advanced techniques like immunohistochemistry, transcriptomics, and proteomics (71). Liver biopsy also remains the gold standard for validating non-invasive diagnostic tools, such as serum fibrosis markers and elastography, by comparing them directly with histological findings to ensure diagnostic reliability (72). In therapeutic trials, particularly those targeting conditions like NASH, fibrosis, or AIH, biopsy is frequently used to assess histological endpoints such as fibrosis regression or reduced inflammation, which are critical markers of treatment response (73). Furthermore, biopsy-derived tissue supports genomic and epigenetic studies, including RNA sequencing, to classify patients based on immune or fibrotic profiles, contributing to the advancement of precision medicine strategies (74). Lastly, liver biopsies are fundamental to human tissue biobanking, where they serve as standardized and well-characterized specimens for longitudinal and multicenter studies, enabling retrospective analyses and the development of consistent histological and molecular scoring systems (75).

Liver Investigation, Testing Marker Utility in Steatohepatitis (LITMUS) is a large-scale European multi-center research consortium (Grant Agreement GA-777377) launched to develop, validate, and qualify non-invasive biomarkers for non-alcoholic fatty liver disease (NAFLD) and non-alcoholic steatohepatitis (NASH), thereby reducing reliance on liver biopsy. They evaluated 17 serum and imaging biomarkers in 966 biopsy-confirmed cases across 13 countries and demonstrated that while no single biomarker fully replaced biopsy (AUCs up to ∼0.90 for advanced fibrosis), many yielded sufficient accuracy to act as prescreening tools (76). A complementary imaging-cohort protocol (the “LITMUS Imaging Study”) is prospectively recruiting patients for paired imaging and biopsy within 100 days to standardize MRI/elastography endpoints against histology (77). The efforts of LITMUS therefore represent a forward-looking shift from invasive tissue sampling toward multi-modal, non-invasive stratification of liver disease, precisely the kind of paradigm transition discussed in this review.

## Artificial intelligence in imaging for liver disease diagnosis

Conventional imaging techniques such as ultrasound, computed tomography (CT), and magnetic resonance imaging (MRI) offer non-invasive ways to evaluate liver pathology (78, 79); however, they are hindered by limitations like interobserver variability and poor sensitivity in detecting early-stage disease (80). The integration of AI into medical imaging is helping to overcome these barriers by enabling automated, objective analysis, enhancing pattern detection, and supporting predictive modeling (81–83). Recent advancements in AI applications have demonstrated strong potential for improving the detection, classification, and quantification of liver diseases (84, 85), with technologies such as deep learning and radiomics pushing the boundaries of diagnostic accuracy and clinical efficiency (86).

AI models have shown promise in differentiating hepatocellular nodular lesions, such as the deep learning tool, for distinguishing well-differentiated HCC in suboptimal biopsies (87), and in predicting treatment response to atezolizumab-bevacizumab in HCC (88). AI is also being applied in MASLD to improve grading reproducibility, assess fibrosis regression, and enhance endpoint identification in clinical trials (89). Despite these advances, implementation faces challenges including high costs, time-intensive slide scanning, and the need for rigorous multi-institutional validation and regulatory approval (90). Nevertheless, AI is expected to support pathologists in delivering more accurate diagnoses and detailed prognostic insights, reinforcing the value of liver biopsy in future clinical care.

## Hardware advancements and computational infrastructure

The successful training and deployment of AI models in liver imaging and histopathology depend heavily on modern hardware accelerators. Graphics Processing Units (GPUs) and Tensor Processing Units (TPUs) have become standard for deep learning model training, substantially reducing computation time for large datasets of digital slides or imaging sequences (91). High-end GPUs such as NVIDIA A100 or equivalent enable parallel processing of millions of image pixels during model optimization (92). For clinical deployment, lightweight inference models are increasingly being implemented on edge-computing devices and AI-enabled scanners, allowing real-time analysis at the point of care without the need for cloud-based processing. These hardware innovations, coupled with advances in data storage, parallel computing, and energy-efficient processors, have made the integration of AI into liver disease diagnostics both practical and scalable in modern healthcare settings (93).

## Ultrasound-based methods

Recent advances in AI have significantly improved the classification and diagnosis of focal liver lesions (FLLs), grading liver steatosis, liver fibrosis, and HCC. Deep learning models, particularly convolutional neural networks (CNNs), have demonstrated high accuracy, up to 96.6%, in staging liver diseases and distinguishing between lesion types, often outperforming traditional machine learning and even experienced radiologists (94–96). Ultrasomics, which combines radiomic feature extraction with machine learning, has shown strong potential in differentiating between primary and metastatic liver tumors, improving the accuracy of preoperative grading and non-invasive diagnosis of HCC and intrahepatic cholangiocarcinoma (ICC) (97, 98). Contrast-enhanced ultrasound (CEUS) has also benefited from AI integration; machine learning and deep learning models applied to CEUS images have enhanced FLL classification, with some models achieving over 90% accuracy and superior diagnostic performance in grading HCC and identifying atypical lesions (99, 100).

A study showed that an extreme learning machine model significantly improved both accuracy and diagnostic speed in detecting fatty liver, outperforming traditional methods like SVM (101). Deep convolutional neural networks (DCNNs) have been widely applied in MASLD assessment, with models showing high accuracy (102, 103). Ensemble and multi-view approaches, such as VGG19 CNN and multi-view US, demonstrated that AI can match or even replace MRI for fat quantification (104–106). Additional methods combining quantitative US, shear wave elastography, and machine learning algorithms like random forests have improved classification of steatosis severity (107, 108). More advanced neural networks, including ResNet-50 v2 and models with AUCs up to 0.99, have demonstrated exceptional performance in classifying varying degrees of liver fat (109, 110). Automated systems have also achieved near-perfect diagnostic accuracy, with some models reaching over 99% and CAD systems reporting 93.33% accuracy in steatosis grading (111, 112). It was developed a DCNN trained on ultrasound data from 3,446 patients, achieving high diagnostic accuracy, which was validated on both internal and external test sets of 266 and 572 patients, respectively (113). Similarly, another study utilized five ultrasound-derived variables, liver parenchyma, spleen thickness, hepatic vein waveform, hepatic artery pulsatility index, and damping index, as inputs to a neural network, resulting in an impressive area under the curve (AUC) of 0.92 for fibrosis diagnosis (114).

## Magnetic resonance imaging-based methods

Recent advancements in 3D CNN architectures and AI techniques have significantly improved the diagnosis and classification of atypical HCC. Studies utilizing multi-sequence MRI, including contrast-enhanced and non-contrast images, have achieved high accuracy (up to 92%) and AUCs exceeding 0.9 in differentiating HCC from non-HCC lesions and other liver tumors (115). AI models like AlexNet and various CNNs have demonstrated performance comparable to or surpassing expert radiologists in lesion grading, detection, and segmentation, with some models achieving accuracies around 90% and AUCs close to 0.95 (116). Incorporating clinical data and advanced image analysis methods such as texture and topological data analysis further enhanced diagnostic precision, often exceeding traditional methods (117). In addition, 3D volumetric analysis using specialized CNNs improved lesion classification accuracy, highlighting AI’s valuable role in early detection, non-invasive assessment, and detailed characterization of liver tumors (118). One model, the radiomics hepatic venous pressure gradient (rHVPG), provides a non-invasive, accurate way to detect cirrhosis and portal hypertension, and has been adapted for use with MRI. In addition, a DCNN applied to gadoxetic acid–enhanced MRI showed strong correlation with biopsy results, with high accuracy in diagnosing different levels of fibrosis (119, 120).

## Computed tomography-based methods

Artificial intelligence has greatly advanced automated tumor segmentation, outperforming traditional methods with high accuracy metrics and reducing false positives through innovative techniques like voxel- and object-level models, as well as adversarial training, achieving performance comparable to manual segmentation (121, 122). It has also been applied to non-invasive tumor grading, helping in risk stratification with models reaching AUCs of up to 0.80, thus aiding in identifying high-risk HCC patients (123, 124). Furthermore, AI has optimized CT imaging protocols to reduce radiation exposure without sacrificing diagnostic accuracy and improved recurrence monitoring by increasing tumor detection rates from 72% to 86% with CNN-based classifiers (125, 126). Various deep learning models have demonstrated high lesion detection accuracy, effective classification of liver tumors, and precise segmentation, with some models achieving near-perfect detection and classification rates, often outperforming or matching radiologists. These advancements underscore AI’s role in enhancing diagnosis, prognosis, and treatment planning in liver cancer imaging (127, 128).

Deep learning-based fibrosis scores from CT images correlate well with liver fibrosis stages, with AUCs around 0.73 to 0.76 for different stages, reflecting strong predictive ability. AI-driven models have enhanced the objectivity and accuracy of fibrosis assessment, evidenced by a deep learning system analyzing contrast-enhanced CT images of thousands of patients, achieving high overall accuracy (79.4%) and AUCs exceeding 0.95 for cirrhosis and advanced fibrosis stages. These advancements demonstrate AI’s potential to refine non-invasive liver fibrosis diagnosis using CT imaging (129, 130). AI-powered methods combined with CT imaging offer a promising, non-invasive approach for diagnosing liver steatosis. Automated liver attenuation analysis has demonstrated high accuracy in detecting MASLD on CT scans (131), while natural language processing algorithms have achieved over 90% recall and accuracy in identifying fatty liver disease across ultrasound, CT, and MRI reports (132). Deep learning models have also been used to assess liver CT values effectively in asymptomatic individuals during routine screenings (133).

This study developed and validated automated classifiers to detect macro- and microsteatosis in digital images of 27 H&E-stained murine liver biopsies using expert annotations, image processing, and supervised machine learning. The macrosteatosis model demonstrated high performance, with 94.2% precision, 95% sensitivity, 99.1% AUROC, and a strong correlation with pathologist grading (*R*^2^ = 0.905), achieving 100% accuracy on unseen samples. The microsteatosis model showed lower performance, with 79.2% precision, 77% sensitivity, 78.1% AUROC, and 63% validation accuracy. These results support the feasibility of fully automated steatosis assessment in murine liver biopsy images, particularly for macrosteatosis (134). A deep learning AI algorithm was developed to quantify fatty vacuoles in mouse liver slides, modeling non-alcoholic fatty liver disease. Using a segmentation framework, it analyzes live histopathology fields during microscope assessments and showed a strong correlation (*r* = 0.87, *P* < 0.001) with manual semiquantitative evaluations, demonstrating its potential to enhance accuracy and efficiency in toxicologic pathology workflows (135).

An automated machine learning algorithm was developed to classify histological features of MASLD, including macrosteatosis and other white regions in H&E-stained liver biopsy images, using annotations from expert pathologists. Analyzing 47 biopsies at 20 × magnification, the classifier achieved 89% overall accuracy, with high precision and recall (≥82%) for macrosteatosis, bile ducts, portal veins, and sinusoids. This accurate detection of liver features supports its potential for clinical and research applications by reducing human variability and enabling precise localization of lesions (136). A novel digital algorithm was developed to accurately quantify hepatic steatosis in whole-slide images of liver tissue, using logistic regression to distinguish steatotic areas based on size and roundness. Validated on samples from 61 MASLD patients and 18 controls, the algorithm achieved an AUC of 0.970% and 91.9% accuracy, with the steatosis proportionate area (SPA) significantly correlating with steatosis grade. This tool, implementable in FIJI software, offers reliable automated assessment for clinical and research applications, including therapeutic trials in MASLD (137).

Several studies have explored modifications to the U-Net architecture to enhance liver and tumor segmentation, often reporting Dice Similarity Coefficient (DSC) as a key metric. A study proposed X-Net, a novel architecture for 2D intra-slice feature extraction of liver and tumors. By incorporating an up-sampling branch and a pyramid-like convolution network into the backbone dense U-Net, the method achieved a DSC of 0.971 on liver and 0.843 on tumor based on the MICCAI 2017 LiTS Challenge dataset, showing high sensitivity and accuracy (138).

To further improve segmentation performance, another study presented a modified U-Net methodology comprising a 58-layer architecture for LiTS. Their model obtained a DSC of 0.9615 on liver and 0.8938 on tumor for the LiTS dataset, and 0.9194 on liver and 0.6980 on tumor for the 3DIRCADb dataset, demonstrating the generalizability of U-Net to different datasets but also highlighting potential challenges in accurate segmentation across diverse data. Though highly relevant, the authors failed to discuss the Specificity metric and the performance impact (139). Moreover, another study introduced a novel architecture called RMS-U-Net, incorporating residual blocks and a multi-scale strategy to deal with inter-slice features with multi-channel input images. The network showed a DSC of 0.9731 on 3DIRCADb, 0.9738 on LiTS, 0.9739 on SLiver07, and 0.9549 on CHAOS datasets for liver segmentation. RMS-U-Net’s consistent performance across various datasets suggests its efficacy in multi-dataset training (140). These research works highlight the effectiveness of U-Net-based approaches involving a U-shaped architecture with skip connections, as it enables a more efficient localization of the area of interest, leading to a better segmentation performance. However, the use of metrics beyond DSC and accuracy, such as those for measures of sensitivity and specificity, should be considered to better the evaluation.

## AI and liver biopsy

Artificial intelligence is emerging as a transformative tool in liver pathology, enhancing the analysis of digital liver biopsy slides by detecting biological and prognostic features beyond human recognition (141). Early applications of AI in liver imaging leveraged established object detection architectures, such as Faster R-CNN. For example, a study demonstrated the use of a modified Faster R-CNN to automate liver tumor detection in abdominal ultrasonography, achieving improved precision and sensitivity compared to the plain Faster R-CNN model (142). In addition, Single Shot Detection (SSD) has shown promise in real-time detection tasks, offering a balance between speed and accuracy, which is crucial for clinical applications.

An AI-based automated tool was developed to detect, quantify, and assess architectural patterns of hepatic fibrosis in trichrome-stained liver biopsies from MASLD patients. Using 987 expert-annotated regions and supervised machine learning, the tool measured collagen proportionate area (CPA), showing good to excellent correlation with pathologists’ fibrosis scores (*R*^2^ = 0.60–0.86). It also accurately identified six fibrosis patterns, with AUROC values ranging from 78.6% to over 90%. This tool offers a reliable method for fibrosis evaluation in both clinical and research settings (143).

The performance and robustness of AI models are heavily reliant on the quality and size of the training dataset. To address these challenges, training data typically undergoes significant preprocessing. Common preprocessing steps include noise reduction, intensity normalization, and standardization to ensure consistent input characteristics (144). Furthermore, data augmentation techniques are often employed to artificially expand the training dataset and improve the model’s ability to generalize to unseen data. These techniques can include geometric transformations such as flipping, rotation, and cropping, as well as intensity adjustments like brightness and contrast manipulation (145). Additionally, adding noise or applying Gaussian blurring can improve robustness to variations in image quality encountered in real-world data (146). By carefully implementing these preprocessing and data augmentation methods, AI models can achieve improved accuracy, reliability, and generalizability in the analysis of liver biopsy images.

A refined AI-based algorithm, qFibrosis, was developed to assist in accurately distinguishing between stage 1 and stage 2 fibrosis in NASH patients, where subtle histological differences can lead to misclassification and exclusion from clinical trials. Using liver biopsies from 160 patients and expert consensus readings, the updated algorithm incorporated 26 novel periportal and perisinusoidal parameters that significantly differentiated true F1 from F2 cases. This enhanced discriminatory ability makes qFibrosis a valuable support tool for pathologists in correctly staging fibrosis and improving patient selection for NASH clinical trials (147).

Second harmonic generation (SHG) microscopy was compared to traditional histological and immunohistochemical methods for visualizing collagen in tissues, demonstrating high specificity for fibrillar collagens. When combined with two-photon excited fluorescence (2PEF), SHG enabled 3D imaging of collagen synthesis and assembly in GFP-expressing transgenic models. The study also introduced scoring methods to quantify collagen accumulation from SHG images, which proved sensitive in detecting renal fibrosis in a murine model through tissue segmentation using endogenous 2PEF signals (148). Liver fibrosis assessment remains essential in MASLD/NASH for predicting outcomes and evaluating treatment response. While non-invasive methods like elastography are preferred, histological evaluation via liver biopsy remains the gold standard. Collagen proportionate area offers continuous quantification but lacks architectural detail. Emerging SHG/TPEF microscopy provides high-resolution imaging of collagen structure, enabling development of AI-supported quantitative fibrosis scores (e.g., qFibrosis) validated against fibrosis stages. Though not yet routine, SHG shows strong potential as a future gold standard, supporting its continued use in clinical trials to enhance evaluation of antifibrotic therapies (149).

Hepatic steatosis (HS) is a key histological feature in many liver diseases, but its conventional assessment remains semi-quantitative and subjective. This study evaluated the use of SHG microscopy with an automated algorithm to diagnose and quantify HS in 86 liver biopsies. SHG showed high reliability among pathologists (ICC = 0.92) and strong correlation with manual assessments (*r* = 0.93, *p* < 0.001), demonstrating its potential as a precise, objective tool for improving histological evaluation of HS (150). HS, a common but often underdiagnosed cardiometabolic risk factor in coronary artery disease patients, was assessed using an AI model that analyzed CT attenuation correction (CTAC) scans from 27,039 patients undergoing myocardial perfusion imaging (MPI). The AI automatically quantified liver and spleen measures to identify HS and developed a liver risk index (LIRI) for enhanced prognostic assessment. HS was detected in 24% of patients and was linked to increased all-cause mortality (adjusted HR 1.14), while LIRI offered stronger predictive value (adjusted HR 1.5). This AI approach enables automated, radiation-free HS detection and improves mortality risk stratification (151).

Approximately one-third of U.S. adults have hepatic steatosis, and coronary artery calcium (CAC) scans may hold untapped diagnostic value. This study applied a novel AI-CVD algorithm to CAC scans from 5,702 asymptomatic individuals in the Multi-Ethnic Study of Atherosclerosis to measure liver steatosis via the liver attenuation index (LAI). Over 15 years, higher LAI was independently associated with increased risk of cardiovascular disease, stroke, and all-cause mortality. These findings demonstrate that AI can opportunistically extract liver fat data from CAC scans, offering early, radiation-free risk stratification for adverse cardiovascular outcomes (152). Metabolic dysfunction-associated steatohepatitis (MASH) is a leading cause of liver-related illness, yet treatment evaluation is hindered by variability in manual biopsy scoring. This multisite study validated AI-based measurement of metabolic dysfunction-associated steatohepatitis (AIM-MASH) an AI-based pathology tool designed to assist histological assessment in MASH clinical trials. AIM-MASH showed high reproducibility and improved accuracy in evaluating key features such as inflammation, ballooning, and MASH resolution, while matching manual performance in steatosis and fibrosis scoring. These results support AIM-MASH as a reliable tool to reduce reader variability and enhance therapeutic assessment in MASH trials (153).

This study developed and validated an AI model to quantify large droplet fat and fat-induced artifact (FIA)/lipopeliosis on preimplantation liver frozen sections, aiming to improve assessment of steatosis, which is linked to delayed graft function. Applied to 161 liver transplant cases, the AI model’s steatosis measurements correlated with early allograft dysfunction, respiratory failure, and advanced fibrosis, but not with overall graft or patient survival. Interestingly, FIA/lipopeliosis levels were associated with graft and patient survival. These findings suggest AI-assisted evaluation may enhance prediction of post-transplant outcomes (154). This study applied an AI system using SHG/two-photon-excited fluorescence (TPEF) imaging to evaluate hepatic steatosis in six MASLD mouse models. While traditional NASH CRN histological scoring failed to detect changes between 8 and 16 weeks, the AI tool objectively quantified dynamic alterations in the area, number, and size of steatotic regions. These results suggest that AI analysis offers a more accurate and sensitive method for assessing steatosis progression in preclinical MASLD models, improving the evaluation of experimental and therapeutic interventions (155).

Steatotic liver disease is the most common chronic liver condition globally, and ultrasonography (US) is widely used for diagnosis. This study developed an AI-based algorithm to improve US diagnostic accuracy by automatically calculating the hepatorenal index (HRIA) and validated it against Proton Density Fat Fraction MRI scans (MRI-PDFF), in 134 patients. The AI-derived HRIA showed stronger correlation with liver fat percentage (*R* = 0.79) and better diagnostic performance (AUC = 0.87) than manually calculated indices (HRIM, *R* = 0.69; AUC = 0.82), with high accuracy in distinguishing steatosis severity. AI enhances the precision and efficiency of US in detecting liver steatosis, supporting its potential for routine clinical use (156). Severe macrovesicular steatosis in donor livers is linked to primary graft dysfunction, prompting the Banff Working Group to propose a standardized three-step approach for assessing large droplet fat (LDF). In this study, 113 donor liver biopsy slides were reevaluated using the Banff method, computer-assisted manual quantification, and an AI model. All samples were confirmed to have <33% LDF, despite initial reports suggesting higher levels in some cases. Strong correlations were observed among the three assessment methods (*r* = 0.81–0.94, *P* < 0.0001), supporting the use of the Banff algorithm and AI for rapid, objective steatosis evaluation in donor livers (157).

Traditional liver disease grading relies on subjective scoring, but this multicenter study of 156 patients evaluated digital pathology’s effectiveness in assessing hepatic inflammation. Using quantitative (I-score) and morphometric (C-score) metrics, digital analysis showed strong correlation with inflammation grades, especially in chronic hepatitis (CH) (ρ = 0.85–0.88) and moderate correlation in MASLD (ρ = 0.5–0.53). CH showed higher inflammation scores than MASLD, and the C-score demonstrated high accuracy (AUC = 0.99) in detecting moderate/severe CH. Overall, digital pathology offers a more objective and accurate tool, particularly for evaluating inflammation in CH (158).

Non-alcoholic steatohepatitis (NASH) is commonly assessed using the semiquantitative NASH Clinical Research Network system, which is limited by interobserver variability. This study developed and validated qFIBS, an automated SHG/TPEF imaging-based algorithm that quantifies four key histological features: fibrosis, inflammation, ballooning, and steatosis, as continuous variables. Using 219 biopsy samples from seven centers, qFIBS showed strong correlations with CRN scores and high diagnostic accuracy, particularly for fibrosis and steatosis. While performance was slightly lower for severe inflammation and ballooning, qFIBS provides a reliable, standardized tool to enhance reproducibility in NASH clinical trial assessments (159). This multicenter study developed and validated a deep learning model, the Autoimmune Liver Neural Estimator (ALNE), to accurately differentiate AIH from primary biliary cholangitis (PBC) using H&E-stained liver biopsy slides. Trained on 354 cases and validated on 92, ALNE achieved strong diagnostic performance (AUC 0.81) without human annotations and outperformed general pathologists, whose agreement was low (Fleiss’s kappa 0.09). The model focused on inflammation-rich regions, associating them with AIH, and misclassified PBC cases often showed overlapping lab values. ALNE offers the first quantitative AI tool for distinguishing between AIH and PBC (89).

Despite remarkable progress, current AI systems face reproducibility and validation challenges. Many reported models rely on small, single-center datasets and lack external validation, which limits generalizability (160). Integration into pathology workflows also demands interoperability with laboratory information systems and adherence to regulatory frameworks. Bridging this translational gap will require multicenter datasets, standardized reporting metrics, and cost-effectiveness analyses demonstrating clear clinical benefit over expert human review (160, 161).

Recent advances in deep learning have produced high-performance tissue segmentation frameworks capable of analyzing complex liver biopsy histopathology with remarkable precision. Among these, Mask R-CNN and YOLOv8 represent two cutting-edge architectures with distinct yet complementary strengths. Mask R-CNN performs instance segmentation by identifying individual histological components such as steatosis droplets, fibrotic septa, or inflammatory foci, generating pixel-level masks that preserve spatial context (162, 163). In contrast, YOLOv8 offers superior speed and real-time detection capacity, enabling rapid localization of diagnostic features with high accuracy. When trained on annotated whole-slide images, these models achieve mean Intersection-over-Union (mIoU) scores exceeding 0.90 and F1-scores above 0.92 for fibrosis and steatosis detection, outperforming earlier U-Net-based models (164). The integration of these architectures into digital pathology pipelines holds great potential for objective quantification, reduced observer variability, and enhanced reproducibility in liver disease grading.

Recent advances in deep learning have also been successfully applied to quantify steatosis in non-tumorous liver tissue. A Mask R-CNN–based model was trained to segment overlapping lipid droplets in histopathological liver images with high precision. By extending Faster R-CNN to include pixel-level mask prediction, the model achieved 75.9% Average Precision, 60.7% Recall, 65.9% F1-score, and a Jaccard Index of 77.0%, effectively distinguishing clustered steatosis regions. This approach demonstrates the capacity of instance segmentation networks to overcome challenges posed by overlapping lipid vacuoles, an essential step for accurate quantification of steatosis burden, fibrosis grading, and assessment of graft quality in transplantation pathology. Such developments emphasize how AI-driven histopathological segmentation extends well beyond hepatocellular carcinoma, offering robust quantitative support in fatty liver disease and transplant evaluation (165).

U-Net and its enhanced variants continue to play a central role in liver biopsy image analysis. A recent study introduced a Multiple Up-sampling and Spatial Attention–guided U-Net (MUSA-U-Net) to automatically segment portal tract regions in whole-slide liver biopsy images. Incorporating depth-wise separable convolution, spatial attention, and residual connections, the model achieved high segmentation performance with an F1-score of 0.89, accuracy of 0.89, and Jaccard Index of 0.80. The quantified portal tract area correlated strongly with clinical fibrosis stage (*R* = 0.681), underscoring the model’s diagnostic relevance. This work highlights how optimized U-Net architectures can provide precise, reproducible quantification of fibrotic features, significantly reducing manual workload and observer variability in fibrosis staging (166).

A YOLOv8-based framework has been developed to enhance accuracy in identifying and classifying liver disease features in digital biopsy images. Trained on a diverse dataset of 3,976 annotated liver images, the model achieved substantial gains in precision, recall, and mean average precision (mAP@0.5), outperforming traditional convolutional and hybrid networks. The YOLOv8 architecture, optimized for medical image analysis, enables rapid object detection and localization while maintaining high diagnostic accuracy. Its integration into digital pathology pipelines demonstrates strong potential for automated lesion detection, fibrosis assessment, and feature quantification in liver biopsy interpretation (164).

The accuracy of AI-based segmentation of liver tissue is highly influenced by hyperparameter tuning during model training (167). Parameters such as learning rate, batch size, and number of epochs directly affect convergence stability, model generalization, and segmentation precision (168). An optimal learning rate ensures efficient gradient updates without overfitting or underfitting, while batch size controls the balance between computational efficiency and model robustness. Similarly, an appropriate number of epochs allows sufficient learning from histopathological features without degrading performance due to excessive training (169). Proper adjustment of these parameters is essential to achieve reliable segmentation outcomes in digital liver pathology.

## Evaluation metrics for segmentation models

The performance of AI-based segmentation models in liver biopsy analysis is primarily assessed using quantitative metrics that reflect accuracy and reliability. The Dice coefficient and Intersection over Union (IoU) are the most critical indicators, measuring spatial overlap between predicted and reference tissue regions (170). Complementary metrics such as precision, recall, and F1-score evaluate the model’s ability to correctly identify histological features while minimizing false detections (171). Together, these measures ensure robust validation of model performance and guide optimization for accurate segmentation of liver architecture, fibrosis, steatosis, and inflammatory regions.

Several AI-based segmentation models have been developed to automate liver biopsy tissue analysis, each utilizing different architectures and validation frameworks. Table 1 summarizes key models, highlighting their datasets, primary applications, evaluation metrics, and diagnostic performance. These developments collectively demonstrate the rapid evolution and clinical potential of AI-assisted liver histopathology.

**TABLE 1:** Comparative summary of segmentation models applied to liver biopsy analysis.

Year	Model/algorithm	Dataset	Application/target feature	Evaluation metrics	Key findings	References
2014	Supervised machine learning classifier	Liver biopsy WSIs (human)	Classification of white regions (steatosis, fibrosis, tissue voids)	Accuracy, precision, recall	Early application of ML for automated tissue classification; demonstrated feasibility but limited by manual feature extraction.	(136)
2018	Texture- and color-based image segmentation	Murine liver tissue	Quantification of steatosis (fat vacuoles)	Correlation with manual grading, MSE	Automated quantification showed high agreement with pathologist assessment; reduced inter-observer variability.	(134)
2019	Digital image processing algorithm	Human NAFLD biopsies (WSI)	Automated steatosis quantification	Pearson’s r, Bland–Altman analysis	Accurate quantification of steatosis; strong correlation with manual pathologist scoring; validated for clinical use.	(137)
2020	qFibrosis (enhanced algorithm)	Human NASH biopsy cohort	Quantitative fibrosis analysis (collagen network segmentation)	AUROC, Correlation with histological stage	Improved precision for fibrosis detection; suitable for patient selection in NASH trials.	(143)
2020	qFIBS (deep learning + feature extraction)	Multicenter NASH biopsy dataset	Quantitative scoring of fibrosis, inflammation, ballooning, and steatosis	R^2^, ICC, AUROC	Fully automated system outperforming conventional semi-quantitative scoring; robust reproducibility.	(158)
2020	CNN for pixel-level steatosis probability maps (VGG16-based)	96 donor liver frozen-section WSIs	Percent steatosis quantification in donor biopsies	*r* = 0.85, ICC = 0.85	DL achieved high reproducibility and speed compared to manual frozen-section reads; clinically relevant for transplant suitability.	(219)
2021	Deep learning–assisted quantitative histology	Human NAFLD biopsies	Comprehensive assessment of fibrosis and steatosis	Accuracy, reproducibility, validation consistency	Review summarizing AI/quantitative advances in fibrosis grading; emphasized histological standardization.	(148)
2024	AIM-MASH (CNN + GNN ensemble)	>16,000 WSIs from six MASH clinical trials	Segmentation and scoring of steatosis, inflammation, ballooning, fibrosis	AUROC, Cohen’s κ, reproducibility	AI matched consensus pathologists with 100% reproducibility; validated for clinical trial endpoint analysis.	(220)
2024	qFibrosis (SHG/TPEF + AI) regional fibrosis metrics	158 paired CHB/CHC biopsies	Quantitative fibrosis dynamics (portal, periportal, midzonal, pericentral)	Regional fibrosis indices, response correlation	AI-DP showed regional fibrosis heterogeneity and treatment response dynamics; enables continuous fibrosis readouts.	(221)
2024	Deep learning for banff consensus steatosis grading	Multicenter donor-liver biopsies	Steatosis assessment aligned with Banff thresholds	Agreement, precision, recall	Automated DL improved standardized donor steatosis grading under Banff consensus framework.	(222)
2025	AI-based pathology platform (deep CNN + ensemble model)	Multicenter MASH trial biopsies	Automated grading of steatosis, inflammation, and ballooning	AUROC, Cohen’s κ, Concordance with experts	Clinically validated AI pathology system showing strong agreement with expert scoring; regulatory-grade robustness.	(152)
2025	SHG/TPEF repeatability and reproducibility	Multicenter SHG/TPEF imaging sets in MASH	Platform validation and QA for quantitative fibrosis imaging	Repeatability and reproducibility statistics	Established platform-level QA framework for quantitative fibrosis imaging in AI-based workflows.	(223)

WSI, whole-slide image; ML, machine learning; MSE, mean squared error; NAFLD, non-alcoholic fatty liver disease; NASH, non-alcoholic steatohepatitis; AUROC, area under the receiver operating characteristic curve; R^2^, coefficient of determination; ICC, intraclass correlation coefficient; DL, deep learning; CNN, convolutional neural network; VGG16, Visual Geometry Group 16-layer network; AIM-MASH, artificial intelligence model for metabolic dysfunction-associated steatohepatitis; GNN, graph neural network; CHB, chronic hepatitis B; CHC, chronic hepatitis C; SHG, second harmonic generation; TPEF, two-photon excitation fluorescence; AI-DP, artificial intelligence–driven pathology; QA, quality assurance; κ, Cohen’s kappa coefficient.

## Multi-omics and liver biopsy

Traditional diagnostic and therapeutic approaches often fall short in capturing the heterogeneity and dynamic nature of these diseases. In recent years, multi-omics technologies, integrating genomics, transcriptomics, proteomics, metabolomics, epigenomics, and microbiome profiling, have emerged as powerful tools to unravel the intricate biological networks driving liver disease pathogenesis and progression (172). By providing a comprehensive, systems-level view of molecular alterations, multi-omics enables precise disease classification, identification of novel biomarkers, understanding of treatment resistance, and development of personalized therapeutic strategies (173).

Many studies explored the application of multi-omics in various liver diseases and clinical contexts. These include uncovering immune and prognostic biomarkers in HCC (174–179), identifying key molecular signatures in early-stage MASLD (180, 181), characterizing the origins and molecular profiles of combined hepatocellular and intrahepatic cholangiocarcinoma (cHCC-ICC) (182), and revealing gut microbiota and metabolite shifts in autoimmune liver diseases such as primary biliary cholangitis (PBC), AIH, and their overlap syndrome (OS) (183). Together, these studies demonstrate the potential of multi-omics to transform liver disease research and management, moving the field closer to precision medicine.

Multi-omics approaches were used to explore the role of Na^+^/K^+^-ATPase subunits in HCC, identifying ATP1B3 as an independently significant prognostic marker. Analyses of transcriptomic and proteomic datasets from TCGA, ICGC, and GEO revealed upregulation of ATP1A1, ATP1B1, and ATP1B3 in HCC, with ATP1B3 particularly associated with poor overall survival and immune-related biological processes. Further investigation showed that ATP1B3 correlates with immune cell infiltration and cytokine expression, and its silencing suppressed HCC cell proliferation and migration while promoting apoptosis and EMT reversal. These findings highlight ATP1B3’s oncogenic potential and its promise as both a prognostic biomarker and therapeutic target in HCC (174). Multi-omics were used to investigate tumor heterogeneity and progression in HCC by analyzing multiple primary cell cultures derived from both primary and recurrent tumors of a single patient. Genomic, epigenomic, and transcriptomic data revealed that while single nucleotide variations (SNVs) showed little functional relevance, copy number alterations (CNAs) and gene body DNA methylation were strongly associated with gene expression differences and phenotypic diversity among tumor subpopulations. These findings suggest that epigenetic and gene dosage changes play a more critical role in tumor behavior than SNVs, highlighting the potential of multi-omics-guided strategies targeting these alterations for personalized cancer therapy (175).

This study explored the use of multi-omics to investigate the prognostic value and molecular characteristics of different gross subtypes of solitary HCC. In a cohort of 400 patients, significant differences in 3-year survival were observed across four gross subtypes, with type IV showing the poorest prognosis. Multi-omics analysis of tumor and non-tumor tissues revealed that type IV tumors had distinct molecular features, including increased angiogenesis, higher immune activity, reduced metabolic pathways, and frequent TP53 mutations. These findings support the use of gross classification, informed by molecular profiling, to guide individualized diagnosis and treatment strategies in HCC (176). This study presents the first longitudinal deep-scale multi-omics analysis, including genomics, transcriptomics, proteomics, and phosphoproteomics, of HCC tumors from eight patients treated with sorafenib. Comparing responders and non-responders, resistance was not linked to a specific mutation but to genomic instability, particularly chromosome 1q gain. Proteomic profiles clearly separated responders from non-responders, with resistant tumors showing features like increased EMT, altered metabolism, and deregulated AMPK and NOTCH signaling. These findings highlight the potential of multi-omics to uncover resistance mechanisms and propose novel biomarkers and combination therapies for improving targeted treatment in HCC (177).

This study used a multi-omics approach, combining whole exome, transcriptome, proteome, and HLA ligandome data, to investigate the presence of naturally presented mutated HLA class I ligands in HCC. Despite identifying somatic mutations, mutated neoepitopes were rarely detected in HCC, in contrast to melanoma, due to HCC’s lower tumor mutational burden. These findings highlight the limitations of relying solely on exome-derived mutations for immunotherapy in low-mutation cancers like HCC and suggest the need to broaden the target range for personalized immunotherapy strategies in such malignancies (178). This study developed and validated a multi-omics-based prognostic model for resectable HCC by integrating genomic, transcriptomic, methylation, and copy number variation data with clinicopathological features. Using data from 330 TCGA patients and 40 external validation cases, key molecular alterations, such as TP53 and FBN1 mutations, CNVs in CSMD1 and RB1, transcriptional changes in AFP and TERT, and methylation changes in genes like DACT2 and PDIA3, were identified as independent survival risk factors. The combined multi-omics model showed high predictive accuracy for overall survival, with AUCs of 0.98 and 0.88 at 1 and 2 years, respectively, offering a powerful tool for personalized prognosis and treatment planning in HCC (179).

This study used a multi-omics approach, combining transcriptomic and metabolomic analyses, to investigate early molecular changes in MASLD in 109 obese individuals. While liver metabolome alterations reflected lipid accumulation, plasma metabolites showed minimal changes. Transcriptomic data revealed distinct gene expression profiles for steatosis and fibrosis, with fibrosis progression marked by global transcriptional changes. Notably, GTPase signaling emerged as a key molecular signature in the transition from steatosis to fibrosis, co-expressed with TGF-β pathways. Functional validation confirmed its role in fibrogenesis, suggesting GTPase modulation as a promising therapeutic target for early-stage MASLD (180). This study used a multi-omics approach, combining 16S rDNA sequencing and targeted metabolomics, to investigate gut microbiota and metabolite changes in children with MASLD. Children with MASLD showed reduced microbial diversity and distinct microbial signatures compared to healthy and obese controls. Notably, Ruminococcus torques was enriched in MASLD patients with high liver stiffness and was positively correlated with deoxycholic acid (DCA) levels and liver disease severity. Mediation analysis suggested that DCA mediates the relationship between R. torques and MASLD progression, highlighting its potential as a therapeutic target for managing pediatric MASLD (181).

**FIGURE 2:**
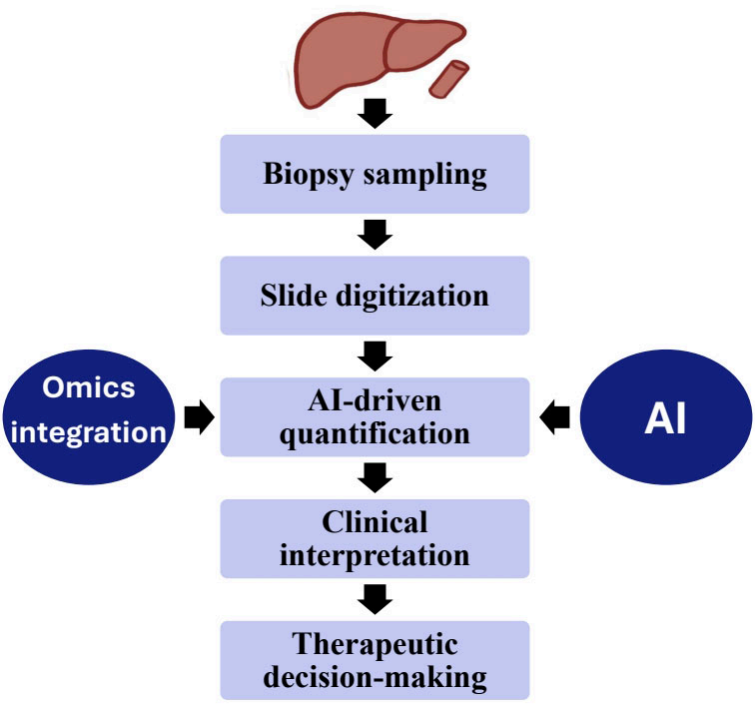
Integration of omics and artificial intelligence in the liver biopsy workflow.

Using multi-omics approaches, this study analyzed 133 cases of combined hepatocellular and intrahepatic cholangiocarcinoma (cHCC-ICC) through genomic and transcriptomic sequencing, revealing distinct clinical and molecular profiles among separate, combined, and mixed subtypes. Integration of laser microdissection, cancer cell fraction analysis, and single nucleus sequencing showed that separate type cHCC-ICCs can arise from both mono- and multiclonal origins, while combined and mixed types are monoclonal. Notably, high Nestin expression was identified across cHCC-ICCs, suggesting its potential as a diagnostic biomarker. These findings provide valuable insights into the classification, origin, and molecular characteristics of cHCC-ICC, aiding future diagnostic and therapeutic strategies (182). This study used a multi-omics approach, combining 16S rRNA sequencing and LC-MS-based metabolomics, to explore the distinct gut microbiota and serum metabolite profiles in autoimmune liver diseases—primary biliary cholangitis (PBC), AIH, and their overlap syndrome (OS). OS patients exhibited significantly reduced microbial diversity and notable taxonomic shifts, along with unique serum metabolites compared to PBC and AIH groups. Correlation analyses linked liver function marker AST with the bacterial genus Fusicatenibacter and the metabolite L-Tyrosine. These findings suggest that multi-omics can uncover underlying mechanisms of OS and support its use in improving diagnosis and stratification in autoimmune liver diseases (183). Figure 2 shows the integration of AI and multi-omics data into the liver biopsy workflow, from sampling and slide digitization to AI-driven analysis and clinical interpretation. The combined morphological and molecular insights enhance diagnostic precision and guide personalized therapeutic decisions.

While multi-omics provides unprecedented molecular depth, integration across data layers remains technically and computationally complex (184, 185). Reproducibility is hindered by variable sample preservation, sequencing depth, and analytical pipelines (186, 187). Translation into clinical use requires validated biomarkers and harmonized bioinformatics standards that can correlate molecular findings with histopathological features (188).

## Single-cell and single-nuclei omics validation of biopsy-derived models

Recent advances in single-cell and single-nuclei RNA sequencing (snRNA-seq), combined with spatial and quantitative proteomics, have revealed novel hepatocellular carcinoma (HCC) subtypes with distinct molecular and immune-microenvironment signatures (189). These integrative approaches validate and extend bulk biopsy–derived multi-omics by resolving intratumoral heterogeneity at cellular resolution and linking it to clinically relevant proteogenomic classes (189). When coupled with AI-driven models, single-cell data can uncover transcriptional and proteomic patterns associated with prognosis, immune evasion, and immunotherapy response (190). This convergence of single-cell and bulk omics strengthens the predictive power of biopsy-based biomarkers and supports refined prognostic models to guide individualized therapies, including checkpoint blockade in HCC (191).

## Feasibility of multi-omics in resource-limited settings

The global implementation of multi-omics approaches faces significant barriers related to high costs, technical expertise, and advanced laboratory infrastructure requirements, particularly in low- and middle-income regions where liver disease burden is greatest (192). Establishing sustainable omics research networks in such settings requires scalable, cost-effective platforms and international collaboration. Emerging solutions such as federated learning frameworks enable multicenter AI model training without centralized data sharing, preserving patient privacy while leveraging diverse datasets (193). Cloud-based bioinformatics tools and open-access omics repositories further lower computational barriers, promoting equitable access to advanced technologies (194). These developments may help bridge the gap between high-resource and low-resource healthcare systems, enabling broader application of precision hepatology worldwide.

## Unified workflow for integrating biopsy histology with AI-analyzed multi-omics data

A standardized workflow is critical for merging histopathological and multi-omics data into a cohesive analytical pipeline. The process begins with specimen-adequacy assessment: for example, liver-biopsy cores should be approximately 2 cm in length and include ≥11–15 complete portal tracts in order to ensure reliable histological and molecular analyses (195). Following fixation and sectioning, digital whole-slide acquisition using high-resolution scanners is performed, and image preprocessing, segmentation, and feature extraction (e.g., fibrosis, steatosis, inflammation) are enabled via AI or machine-learning methods (196). In parallel, multi-omics profiling (genomic, transcriptomic, proteomic, metabolomic) is conducted on validated platforms, with stringent quality-control steps such as RNA-integrity checks, sequencing-depth thresholds, and batch-normalization to ensure data reliability. The data-integration phase employs machine-learning or deep-learning “fusion” models that correlate morphological image features with molecular signatures, demonstrated in modalities coupling histopathology with omics for prognostic modeling (197). The final stage involves model validation, interpretability, cross-center harmonization protocols and standardized reporting to ensure reproducibility across institutions (198). This unified framework thereby establishes a scalable approach for precision liver-biopsy analytics across research and clinical environments.

## Integrative framework: from morphology to molecular insight

Traditional histopathology captures architectural and cytological context, while artificial intelligence (AI) quantifies patterns beyond human perception and multi-omics reveals molecular pathways underpinning those features (196, 199, 200). When combined, these layers transform liver biopsy from a purely morphological tool into a multidimensional diagnostic platform. For instance, AI-quantified fibrosis metrics can be directly correlated with transcriptomic and proteomic profiles to stratify patients by molecular subtype and treatment response (201, 202). This integrative model supports a precision-hepatology paradigm where histological, digital, and molecular data converge to inform individualized management (196, 202).

## AI-driven omics integration and personalized therapy

The fusion of multi-omics data, such as genomics, transcriptomics, proteomics, and metabolomics, through AI algorithms enables the identification of complex molecular signatures predictive of therapeutic response (203). In hepatocellular carcinoma (HCC), AI-driven models integrating transcriptomic and proteomic data from biopsy samples have revealed immune-related biomarkers and tumor microenvironment features associated with differential response to immune checkpoint inhibitors (204). By learning from multi-dimensional datasets, these systems can stratify patients into molecular subgroups likely to benefit from immunotherapy or targeted treatments (204). Such integrative approaches support precision oncology by transforming biopsy-derived molecular data into actionable clinical insights, thereby optimizing treatment selection and improving patient outcomes.

## Limitations of AI

Despite growing excitement around AI in healthcare, its implementation faces several challenges. Biases may arise when models are trained on single-center datasets with inconsistent quality, limiting their accuracy and generalizability. AI systems, particularly large language models, also risk generating false but convincing outputs, that can endanger patient care without proper human oversight (205). Ethical concerns include potential loss of patient autonomy, susceptibility to manipulation, and conflicts between commercial interests and patient benefit, highlighting the need to uphold principles of non-maleficence, beneficence, and justice (206). Ensuring equitable access, protecting patient data under local regulations, and clearly communicating data use and storage practices are essential for maintaining confidentiality (207, 208). To improve fairness and reliability, AI models should be trained on large, diverse, multi-center datasets and validated extensively on well-characterized patient cohorts before clinical use (209). A key limitation to the widespread adoption of AI tools is their cost and practicality, particularly in resource-limited settings. Models relying on complex omics data or advanced imaging technologies face significant challenges in under-resourced healthcare environments (210). Without the development of lightweight, affordable models that utilize commonly available clinical data, such as liver function tests and anthropometric measures, scalability remains limited. In addition, the lack of seamless integration into existing healthcare infrastructures further hinders the implementation of AI-based solutions (210). Compared with non-invasive imaging biomarkers such as transient elastography or MRI-based radiomics, AI-enhanced biopsy retains the advantage of molecular and architectural resolution but at higher procedural cost and patient risk (211, 212). However, when combined with digital and omics-based analytics, biopsy delivers multi-layered diagnostic and prognostic information that remains unmatched by imaging alone (170, 196). Future clinical validation studies should evaluate hybrid models that combine non-invasive screening with targeted AI-assisted biopsy to optimize accuracy, safety, and cost-effectiveness across diverse healthcare settings (170).

One of the major limitations of AI-assisted histopathology is the occurrence of false positives and segmentation errors, particularly in regions with overlapping histological features or poor staining quality (213). Models trained on limited or homogeneous datasets may misclassify inflammatory foci, fibrotic septa, or steatotic vacuoles, resulting in inflated performance metrics that do not generalize across populations (214). While few segmentation-specific studies report a consistent error-rate range of 5%–15%, classification-oriented surveys indicate significant variability depending on image resolution, staining variability and annotation quality (160). These inaccuracies underscore the importance of incorporating robust validation datasets, human-in-the-loop review processes, and continuous retraining to ensure that automated systems complement, rather than replace, expert pathologists (213).

## Liquid biopsy in liver disease

Liquid biopsy is an evolving non-invasive diagnostic approach that provides valuable molecular insights without the need for tissue extraction. By analyzing circulating biomarkers such as cell-free DNA (cfDNA), circulating tumor DNA (ctDNA), RNA fragments, and extracellular vesicles (including exosomes), liquid biopsy enables dynamic monitoring of liver disease progression, therapeutic response, and recurrence. Recent advances in high-throughput sequencing and proteomics have expanded its role in identifying genomic and epigenomic alterations associated with hepatocellular carcinoma (HCC) and non-alcoholic steatohepatitis (NASH). Although liver tissue remains indispensable for architectural and histological assessment, liquid biopsy offers a complementary strategy for longitudinal disease tracking and early detection, representing a promising step toward minimally invasive precision hepatology (215, 216).

Beyond traditional liquid biopsy applications, multi-omics profiling of circulating biomarkers, including ctDNA, cfRNA, and exosome-derived proteomic and metabolomic signatures, offers a transformative potential to reduce dependence on invasive biopsy. These circulating analytes can capture genomic mutations, methylation patterns, and pathway-level alterations reflective of underlying hepatic pathology and tumor heterogeneity. Integration of such liquid biopsy-derived omics with AI-assisted imaging and digital pathology could establish a composite, non-invasive diagnostic framework capable of longitudinal disease surveillance and therapeutic stratification. Early multi-omics studies in hepatocellular carcinoma and NASH have shown encouraging accuracy in predicting histological grades and fibrosis stages, supporting a gradual shift toward minimally invasive molecular diagnostics (217, 218).

## Conclusion

Liver biopsy continues to be an indispensable tool in hepatology, providing crucial diagnostic and prognostic information. While traditional biopsy methods are well established, they present procedural limitations and interpretative variability. The integration of AI and multi-omics approaches represents a transformative step toward more objective, reproducible, and comprehensive liver disease assessment. AI has demonstrated promise in enhancing the evaluation of imaging and histological features, while multi-omics can elucidate molecular mechanisms underlying disease heterogeneity. Together, these innovations have the potential to refine diagnostic pathways, improve clinical trial enrollment, and support personalized treatment strategies. Future efforts should focus on standardizing AI models, validating multi-omics data, and ensuring equitable access to these technologies in routine clinical practice.
